# A Study on Hemodynamic and Brain Network Characteristics During Upper Limb Movement in Children with Cerebral Hemiplegia Based on fNIRS

**DOI:** 10.3390/brainsci15101031

**Published:** 2025-09-24

**Authors:** Yuling Zhang, Yaqi Xu

**Affiliations:** 1 School of Health Science and Engineering, University of Shanghai for Science and Technology, Shanghai 200093, China; 19962614273@163.com; 2 Shanghai Engineering Research Center of Assistive Devices, Shanghai 200093, China

**Keywords:** cerebral palsy, functional near-infrared spectroscopy, mirror therapy, brain activation, effective connections

## Abstract

**Background**: Hemiplegic cerebral palsy (HCP) is a motor dysfunction disorder resulting from perinatal developmental brain injury, predominantly impairing upper limb function in children. Nonetheless, there has been insufficient research on the brain activation patterns and inter-brain coordination mechanisms of HCP children when performing motor control tasks, especially in contrast to children with typical development(CD). **Objective**: This cross-sectional study employed functional near-infrared spectroscopy (fNIRS) to systematically compare the cerebral blood flow dynamics and brain network characteristics of HCP children and CD children while performing upper-limb mirror training tasks. **Methods:** The study ultimately included 14 HCP children and 28 CD children. fNIRS technology was utilized to record changes in oxygenated hemoglobin (HbO) signals in the bilateral prefrontal cortex (LPFC/RPFC) and motor cortex (LMC/RMC) of the subjects while they performed mirror training tasks. Generalized linear model (GLM) analysis was used to compare differences in activation intensity between HCP children and CD children in the prefrontal cortex and motor cortex. Finally, conditional Granger causality (GC) analysis was applied to construct a directed brain network model, enabling directional analysis of causal interactions between different brain regions. **Results**: Brain activation: HCP children showed weaker LPFC activation than CD children in the NMR task (t = −2.032, *p* = 0.049); enhanced LMC activation in the NML task (t = 2.202, *p* = 0.033); and reduced RMC activation in the MR task (t = −2.234, *p* = 0.031). Intragroup comparisons revealed significant differences in LMC activation between the NMR and NML tasks (M = −1.128 ± 2.764, t = −1.527, *p* = 0.025) and increased separation in RMC activation between the MR and ML tasks (M = −1.674 ± 2.584, t = −2.425, *p* = 0.031). Cortical effective connectivity: HCP group RPFC → RMC connectivity was weaker than that in CD children in the NMR/NML tasks (NMR: t = −2.491, *p* = 0.018; NML: t = −2.386, *p* = 0.023); RMC → LMC connectivity was weakened in the NMR task (t = −2.395, *p* = 0.022). **Conclusions**: This study reveals that children with HCP exhibit distinct abnormal characteristics in both cortical activation patterns and effective brain network connectivity during upper limb mirror training tasks, compared to children with CD. These characteristic alterations may reflect the neural mechanisms underlying motor control deficits in HCP children, involving deficits in prefrontal regulatory function and compensatory reorganization of the motor cortex. The identified fNIRS indicators provide new insights into understanding brain dysfunction in HCP and may offer objective evidence for research into personalized, precision-based neurorehabilitation intervention strategies.

## 1. Introduction

Cerebral palsy (CP) ranks among the most disabling neurodevelopmental movement disorders in childhood, primarily caused by non-progressive brain injury during fetal or infant development, resulting in impaired motor control [1,2]. Upper limb dysfunction, one of the most prominent clinical manifestations in children with CP, severely limits their ability to perform activities of daily living and participate in social interactions [3,4,5]. Epidemiological data indicate that approximately 2 to 2.5 cases per 1000 children occur globally, with a prevalence rate of about 2.46% in China [6,7]. Currently, clinical diagnosis primarily relies on structural magnetic resonance imaging (MRI), which can visually demonstrate characteristic pathological changes such as periventricular white matter lesions and basal ganglia abnormalities [8]. AI prediction models based on MRI data can identify cerebral palsy risk in high-risk infants at an early stage [9]. However, MRI primarily reflects static structural abnormalities and struggles to dynamically capture brain functional activity and network interaction characteristics during task performance. While other auxiliary methods (such as kinematic analysis and electrophysiological monitoring) can assess limb function, their ability to elucidate cortical neural activity mechanisms remains limited. Against this backdrop, fNIRS offers real-time monitoring of brain function to deepen understanding of HCP’s neural mechanisms. While traditional rehabilitation strategies like neurodevelopmental therapy and task-oriented functional training effectively improve upper limb function, their underlying mechanisms for producing neuro-rehabilitative effects remain incompletely elucidated [10]. In contrast, mirror training promotes neuro-rehabilitation by activating mirror neuron systems and enhancing visuomotor integration, thereby more effectively facilitating neural remodeling and functional reorganization [11]. Therefore, investigating the neural rehabilitation mechanisms underlying mirror training interventions in children with HCP is crucial for developing more precise and effective rehabilitation strategies.

In recent years, fNIRS has emerged as a powerful tool for non-invasively investigating brain activity. Due to its unique advantages—including non-invasiveness and strong resistance to motion artifacts—fNIRS is particularly well-suited for studying populations of children who are hyperactive or have difficulty sustaining attention [12,13,14,15,16]. By detecting changes in cerebral HbO and deoxyhemoglobin (HbR) concentrations, this technology reflects hemodynamic responses triggered by local neural activity. It has demonstrated significant value in studying brain function across neurodevelopmental disorders such as autism spectrum disorder and attention-deficit/hyperactivity disorder (ADHD) [17,18,19,20]. Particularly for HCP research, fNIRS overcomes MRI’s static limitations by simultaneously capturing task-related activation patterns and network connectivity dynamics between the prefrontal cortex and motor cortex. This enables deeper understanding of HCP’s neural mechanisms, functional compensation, and rehabilitation biomarkers [21,22,23,24,25]. However, the brain activation patterns and inter-regional coordination mechanisms during upper limb motor tasks in HCP children remain unclear to date.

This study aims to utilize fNIRS technology to simultaneously collect cortical hemodynamic signals from children with HCP and CD children during standardized upper limb mirror training tasks, integrating brain activation analysis with effective connectivity modeling. By systematically comparing differences in cortical activation patterns between the two groups and constructing directed brain network models to analyze their causal interaction characteristics, we seek to reveal the neural mechanisms underlying motor control impairments in children with HCP. The findings are expected to provide novel insights into the neuropathological mechanisms of upper limb dysfunction in HCP children and offer biomarkers for future neuroimaging-based personalized rehabilitation assessment, treatment efficacy prediction, and intervention strategies.

## 2. Materials and Methods

### 2.1. Selection and Grouping of Research Subjects

**Figure 1:**
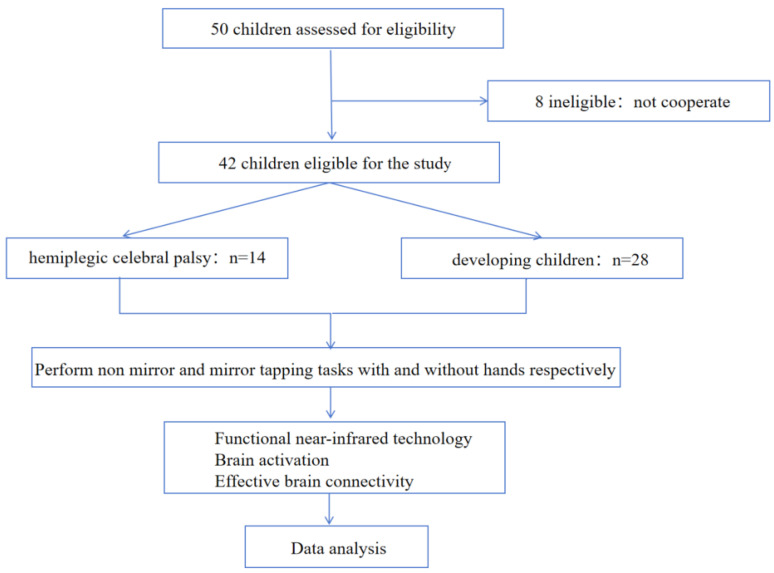
Overall research process diagram.

This study enrolled 20 HCP children who were diagnosed at Shanghai Jiao Tong University Affiliated Xinhua Hospital and Chongqing Medical University Affiliated Children’s Hospital between March 2024 and April 2025, and included them in the HCP group, as shown in Figure 1. Inclusion criteria: (1) Age 3–14 years; (2) Clinically diagnosed with cerebral palsy confirmed by MRI, with right-handedness; (3) No metallic head implants, skull defects, or extensive scarring; (4) Able to comprehend experimental task instructions and maintain basic attention span (≥5 min); (5) GMFCS Level I and capable of completing the experiment independently; (6) Written informed consent signed by a legal guardian. Exclusion Criteria: (1) Severe cognitive impairments or behavioral issues, unable to maintain a resting state or perform standardized movements; (2) History of cranial surgery, cranial trauma, or severe visual/hearing impairments; (3) Head circumference too small resulting in channel coverage < 80%; (4) Received botulinum toxin injections or other interfering treatments within the past 3 months prior to the study.

Concurrently, 30 CD children matched for age and gender were recruited and included in the CD group. Inclusion criteria: (1) Children aged 3–14 years with normal development; (2) Understanding and signing of the informed consent form; (3) Right-handedness. Exclusion criteria: (1) Presence of behavioral issues such as attention deficit or sensory integration dysfunction; (2) History of neurological disorders such as head trauma or encephalitis; (3) Inability to cooperate with examinations or assessments.

This research protocol was approved by the Medical Ethics Committee of Xinhua Hospital Affiliated to Shanghai Jiao Tong University. All participants in the study obtained guardian consent and voluntarily signed informed consent forms.

### 2.2. Basic Information About the Subjects

Fifty children initially participated in this cross-sectional study. However, due to low compliance among some children, eight children were excluded from the study. The final analysis included 14 participants classified as GMFCS Level I by HCP, with an average age of 8.34 years [standard deviation (SD) 1.495 years], an average height of 140.55 cm (SD = 17.02 cm), and an average weight of 37.342 kg (SD = 16.5214 kg). Twenty-eight age-matched CD children were included, with an average age of 7.5 years (SD = 2.726 years), an average height of 115.5 cm (SD = 40.319 cm), and an average weight of 44.186 kg (SD = 37.1998 kg). The characteristics and clinical features of the participants are summarized in Table 1. The sample size for this study was determined based on the feasibility of actual recruitment and reference to previous fNIRS studies in pediatric HCP patients. The sample size is relatively small, so this study serves as an exploratory pilot study, with the primary aim of laying the groundwork for formal efficacy analysis in future larger-scale studies.

### 2.3. Experimental Equipment and Parameter Settings

**Figure 2:**
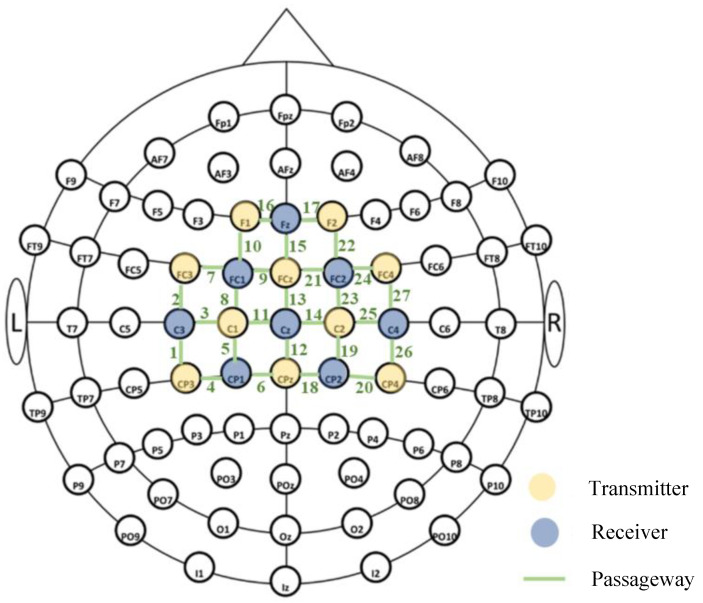
Schematic diagram of fNIRS data acquisition.

Brain functional activity blood oxygenation data were acquired using a multi-channel continuous fNIRS device (Brite24, Artinis Medical Systems, Netherlands) at a sampling frequency of 25 Hz. The system employed near-infrared light sources at wavelengths of 760 nm and 850 nm. It comprised 10 light-emitting diode (LED) emitters and 8 photodiodes, collectively forming 27 measurement channels. Prior to the experiment, corresponding cap sizes were selected based on the children’s head circumferences. Channels were assigned to four regions of interest (ROIs) based on the Montreal Neurological Institute standard template and Brodmann areas: LPFC (left prefrontal cortex), RPFC (right prefrontal cortex), LMC (left motor cortex), and RMC (right motor cortex). The correspondence between fNIRS acquisition channels and brain ROIs, along with channel coordinate locations, is shown in Figure 2 and Table 2, respectively.

### 2.4. Task Paradigm and Testing Process

This study employed a 2 × 2 factorial design. First, participants were grouped based on their condition type and corresponding task direction: HCP children were divided into a task group based on training with the unaffected side and a task group based on training with the affected side; CD children were divided into a task group based on training with the dominant hand and a task group based on training with the non-dominant hand. Second, all participants underwent two visual direction tasks: non-mirrored normal visual feedback task and mirrored visual feedback task. This resulted in four experimental conditions as controls: HCP children conditions: healthy side hand tapping non-mirrored task (NMR), affected side hand tapping non-mirrored task (NML), healthy side hand tapping mirrored task (MR), and affected side hand tapping mirrored task (ML). CD children condition: dominant hand tapping non-mirrored task (NMR), non-dominant hand tapping non-mirrored task (NML), dominant hand tapping mirrored task (MR), and non-dominant hand tapping mirrored task (ML).

**Figure 3:**
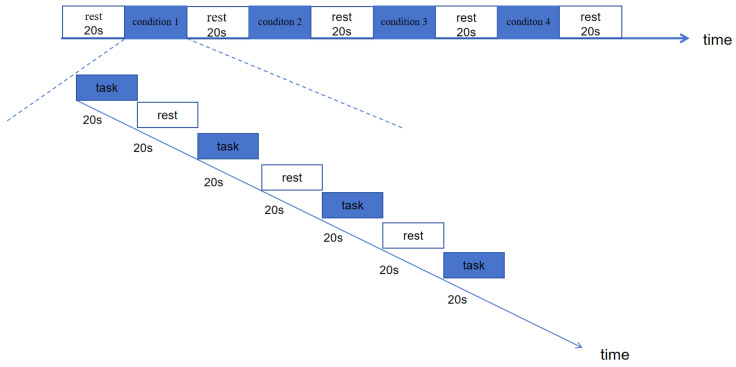
Experimental paradigm diagram.

Task execution follows the standard spoon tapping paradigm, requiring the non-operating hand to remain stationary. Under mirror therapy conditions, participants sit with both arms placed on the table, with a 30 cm × 30 cm mirror positioned vertically along the midline. Participants observe the mirror image of the unaffected/dominant hand to perform motor imagery. The therapist first demonstrates and assists in achieving bilateral movement synchrony, with movement frequency strictly controlled at 0.33 Hz. fNIRS data were collected using a block paradigm. Each condition was initiated after confirming signal baseline stability and included three consecutive blocks. A single block consisted of a 20-s task period and a 20-s rest period: the task period was triggered by a 1 kHz pure tone prompt (lasting 50 ms) via E-Prime 3.0 software to initiate spoon tapping by the operative hand; The rest period requires participants to fixate on a white crosshair target at the center of the display screen (black background) while maintaining body stillness throughout. The task sequence is presented in a pseudo-random order to avoid practice effects, with a 20-s buffer period between conditions to ensure physiological signals return to baseline levels. Data collection is conducted in a soundproof electromagnetic shielded room with environmental illuminance consistently controlled at 200 lux. The experimental paradigm is illustrated in Figure 3.

### 2.5. Data Processing and Analysis

#### 2.5.1. fNIRS Preprocessing

Raw fNIRS data were standardized and preprocessed using the Homer2 software (v2.8, p2.1) package to eliminate noise, motion artifacts, and physiological interference, and to extract task-related hemodynamic response signals. The specific workflow is as follows: 1. Based on the modified Beer-Lambert law, the raw light intensity data from the two wavelengths (690 nm and 830 nm) were converted into changes in optical density (ΔOD); 2. Automatic motion artifact detection is performed on the ΔOD time series for each channel, followed by correction using principal component analysis (PCA). This algorithm adaptively determines the number of principal components based on the criterion of minimizing residual energy to optimize artifact removal; 3. The corrected ΔOD data are processed through a fourth-order Butterworth zero-phase bandpass filter to remove low-frequency baseline drift and high-frequency physiological noise; 4. The filtered ΔOD data are converted into concentration changes of ΔHbO, ΔHbR, and total hemoglobin (ΔHbT) using the differential path factor (DPF) calculation (units: μmol/L); 5. Quality control is performed, including removing low-quality channels with a signal-to-noise ratio (SNR) < 10 dB and excluding abnormal data segments with cross-wavelength signal correlation (|r| < 0.7); 6. Z-score standardization is performed on the ΔHbO and ΔHbR time series using the mean and standard deviation of the pre-task resting period (−5 s to 0 s) as a reference, enabling normalized comparisons across subjects.

#### 2.5.2. Brain Activation Analysis

This study employed GLM to quantify the statistical significance of changes in task-related HbO signal concentration. Specifically, for each channel, the time series of HbO concentration changes during the task relative to the resting baseline (−5 s to 0 s) was modeled using GLM, and the regression coefficient (β value) representing the average activation intensity of that channel under task conditions was obtained. Subsequently, to identify task-related significantly activated brain regions, a one-sample t-test was performed on the β values for each channel (with the null hypothesis that β = 0), and multiple comparisons were corrected using the False Discovery Rate (FDR) method. Channels with corrected *p*-values less than 0.05 were deemed to be significantly activated during the task condition. Finally, this study extracted and reported two core activation metrics: (1) Number of significantly activated channels: the number of fNIRS channels identified as significantly activated through the aforementioned statistical tests, representing the spatial activation range related to the task; (2) Average β value of significantly activated channels: the average of all β values of significantly activated channels, serving as a quantitative indicator of the overall activation intensity of task-related brain regions.

#### 2.5.3. Cortical Connectivity Analysis

To mitigate the influence of functional lateralization on the analysis, the fNIRS data of all participants were uniformly mirror-flipped during the preprocessing stage according to the main task attributes. Subsequently, the effective connectivity toolbox in the HERMES (2019) software platform was used to calculate the directional causal interactions between different ROIs during task execution. Specifically, the GC algorithm was applied within a predefined task-related time window to quantitatively assess the extent to which the past time series information of one ROI enhances the predictive ability of another ROI’s current state, thereby quantifying the directionality and strength of directed connections between brain regions. Original GC values underwent Fisher-Z transformation to improve the normality and additivity of their distributions for subsequent statistical analysis. The transformed values were defined as the effective connection strength between corresponding ROIs, representing the causal influence intensity of neural information flow in a specific direction.

### 2.6. Statistical Analysis

All statistical analyses were performed using SPSS 27.0 and MATLAB R2021a software platforms. For the two core indicators of brain activation intensity and effective connectivity strength, the following methods were employed: brain activation intensity was represented by the regression coefficients (β values) of each ROI region under task conditions calculated using GLM; Effective connectivity strength was assessed using multivariate Granger causality analysis, with the original GC values transformed using the Fisher-Z transformation to improve normality before quantification. Continuous variable data were first assessed for normality and homogeneity of variances between groups using the Shapiro-Wilk test and Levene’s test, respectively. Quantitative data meeting the assumptions of normality and homogeneity of variances were expressed as mean ± standard deviation (M ± SD). Intra-group comparisons were performed using paired-sample *t*-tests to control for inter-individual variability; inter-group comparisons were performed using independent-sample *t*-tests. If data did not meet the assumptions for parametric tests, inter-group comparisons were performed using the Mann-Whitney U test, and intra-group comparisons were performed using the Wilcoxon signed-rank test. Given the multiple comparisons involving multiple ROIs and task conditions, *p*-values for all statistical tests were corrected using the Bonferroni method to control for Type I errors. The significance level was set at α = 0.05, and corrected *p*-values less than 0.05 were considered statistically significant. All statistical results will report specific statistical measures and corrected *p*-values.

## 3. Results

### 3.1. Brain Activation Differences

This study applied GLM analysis of fNIRS data to extract the β coefficient of HbO concentration to quantify the intensity of neurovascular responses within each ROI under task conditions. To test for differences in activation between tasks, statistical inference analysis was performed on the mean β values of each ROI channel, reporting mean differences, t-values, and *p*-values after multiple comparison correction. This framework provides quantitative evidence for interpreting the specificity of blood oxygen responses in brain functional areas under different tasks and group conditions.

#### 3.1.1. Activation Status of Each ROI Based on β Values (Intra-Group Comparison)

##### HCP Group

**Figure 4:**
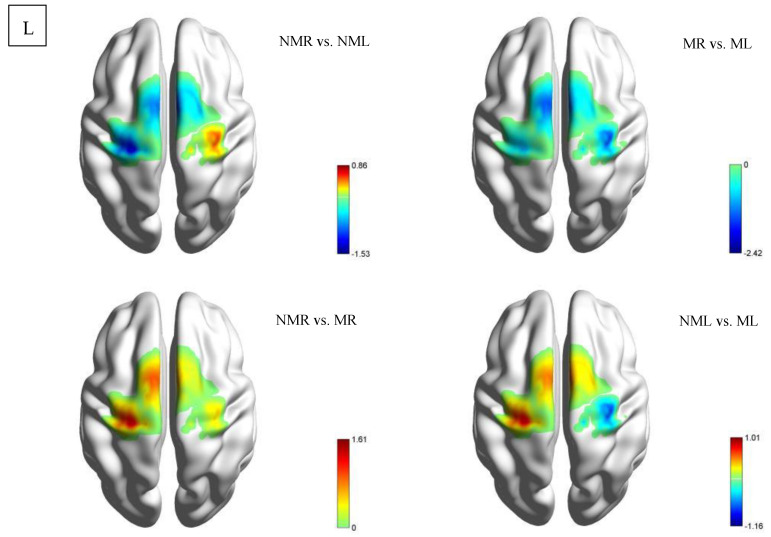
Intra-group comparison of activation in the HCP group. Among these, NMR de notes the normal vision right-hand task; NML denotes the normal vision left-hand task; MR denotes the mirror vision right-hand task; ML denotes the mirror vision left-hand task. L denotes the left view.

This study used a paired samples *t*-test to analyze neural activation differences in the HCP group under four task conditions, as shown in Table 3 and Figure 4. In the comparison between the normal visual left-hand and right-hand tasks (NMR vs. NML), the LMC showed significant activation differences (M = −1.128 ± 2.764, t = −1.527, *p* = 0.025), while other brain regions did not reach significance (LPFC: M = −1.008 ± 4.095, *p* = 0.374; RPFC: M = −3.651 ± 11.942, *p* = 0.273; RMC: M = 2.685 ± 11.699, *p* = 0.406). Notably, the mirrored visual left-right hand comparison (MR vs. ML) showed a significant reduction in RMC activation (M = −1.674 ± 2.584, t = −2.425, *p* = 0.031), with no statistical differences in other brain regions. Cross-visual modality comparison: Right-hand task (NMR vs. MR): No significant differences were observed in any brain region (*p* > 0.05), but there was a clear trend toward enhanced LMC activation (M = 1.177 ± 2.728, t = 1.613, *p* = 0.131). Left-hand task (NML vs. ML): Although RMC showed the largest negative change (M = −3.447 ± 11.12, t = −1.16, *p* = 0.267), the standard deviation was as high as 11.12, rendering the result statistically insignificant.

##### CD Group

**Figure 5:**
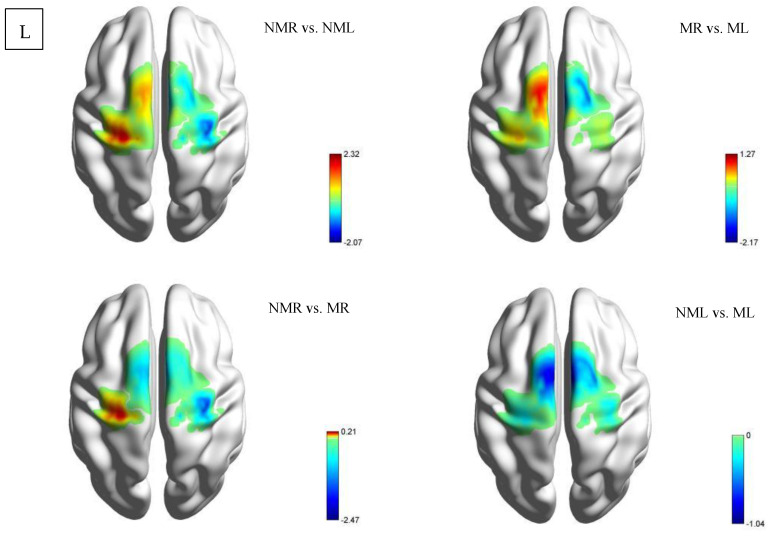
Intragroup comparison activation map for the CD group. Among these, NMR de notes the normal vision right-hand task; NML denotes the normal vision left-hand task; MR denotes the mirror vision right-hand task; ML denotes the mirror vision left-hand task. L denotes the left view.

This study conducted a paired-sample t-test analysis of brain region activation intensity in the CD group, focusing on comparing four conditions: NMR vs. NML, MR vs. ML, NMR vs. MR, and NML vs. ML, as shown in Table 4 and Figure 5. The brain regions included the left/right prefrontal cortex (LPFC/RPFC) and motor cortex (LMC/RMC). The results showed that under normal vision, NMR significantly enhanced activation in the left motor cortex (LMC: t = −2.068, *p* = 0.04, M ± SD = −0.244 ± 4.492) compared to NML; while there was no significant change in the RMC (t = −1.428, *p* = 0.16, M ± SD = −1.405 ± 5.312). Under mirrored vision: the contralateral pattern in the motor cortex disappears (MR vs. ML: LMC: t = 0.605, *p* = 0.55, M ± SD = 0.618 ± 5.503; RMC: t = 0.255, *p* = 0.80, M ± SD = 0.336 ± 7.090). The MR vs. ML condition induced significant activation in the right prefrontal cortex (RPFC: t = −2.173, *p* = 0.03, M ± SD = −1.648 ± 4.085).

#### 3.1.2. Activation Status of Each ROI Based on β Values (Between-Group Comparison)

**Figure 6:**
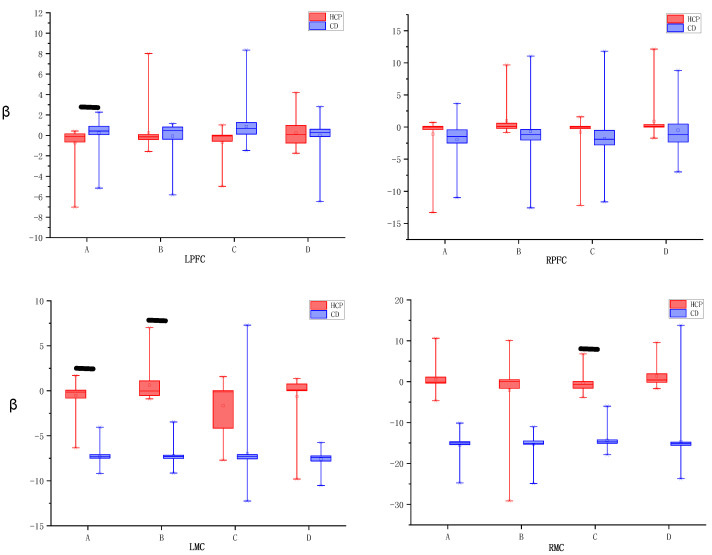
β values between HCP and CD groups ROIs for different tasks, black lines indicate *p* < 0.05.

For comparisons of between-group differences, an independent samples t-test was used, as shown in Figure 6, where A: Normal right-hand task (NMR); B: Normal left-hand task (NML); C: Mirrored visual right-hand task (MR); D: Mirrored visual left-hand task (ML). This method assessed group differences in β values of specific ROIs between HCP and CD group participants when performing the same task, revealing the systematic effects of experimental conditions on brain functional responses. The results revealed neural activation differences between the HCP and CD groups in visual-motor tasks. In the LPFC region, the HCP group exhibited significantly reduced activation in the NMR condition (t = −2.032, *p* = 0.049), and this phenomenon persisted in the MR condition (t = −2.097, *p* = 0.042). The LMC, however, exhibited task-specific patterns: the experimental group showed significantly enhanced activation in the NML (t = 2.202, *p* = 0.033), while the NMR showed marginally reduced activation (t = −2.019, *p* = 0.05). Notably, in the right hemisphere, RMC showed significantly reduced activation in the HCP group under MR conditions (t = −2.234, *p* = 0.031). However, no significant between-group differences were observed in ML and RPFC under mirror vision conditions (*p* > 0.05).

### 3.2. Direction and Coupling Strength of Effective Connectivity in the Cortex

#### 3.2.1. Connection Direction

**Figure 7:**
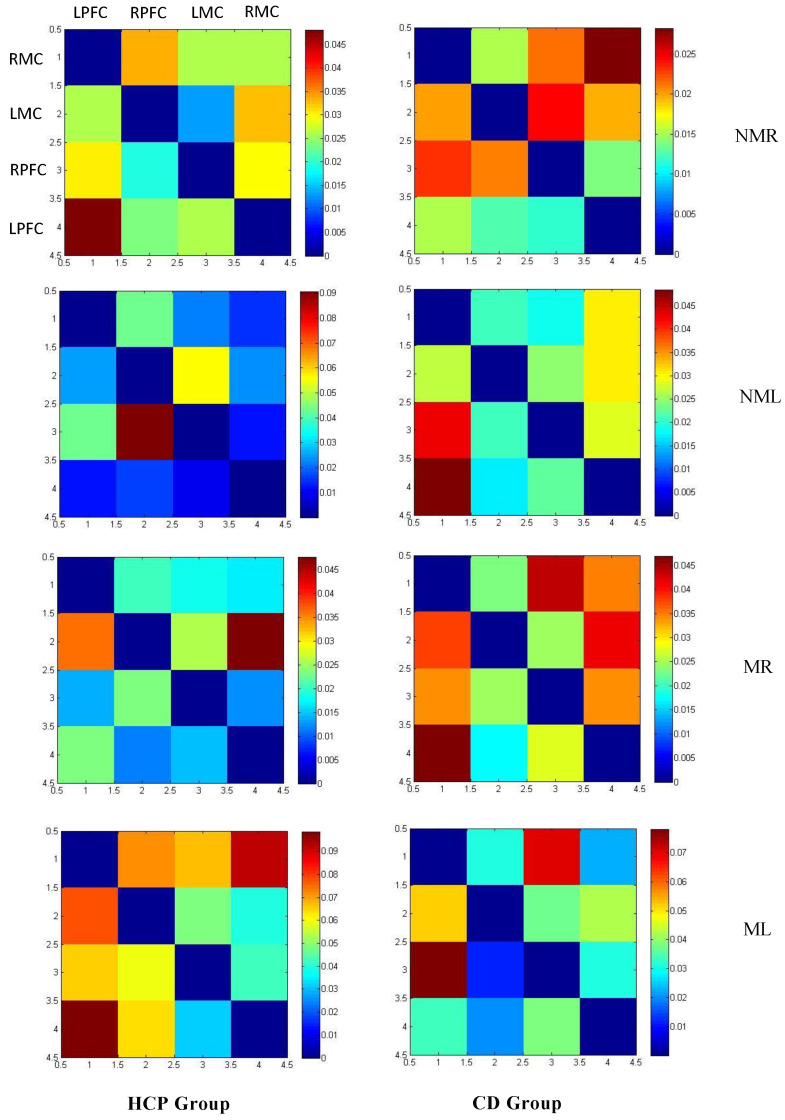
Overall average matrix of GC values for the HCP group and CD group. Among these, NMR de notes the normal vision right-hand task; NML denotes the normal vision left-hand task; MR denotes the mirror vision right-hand task; ML denotes the mirror vision left-hand task.

**Figure 8:**
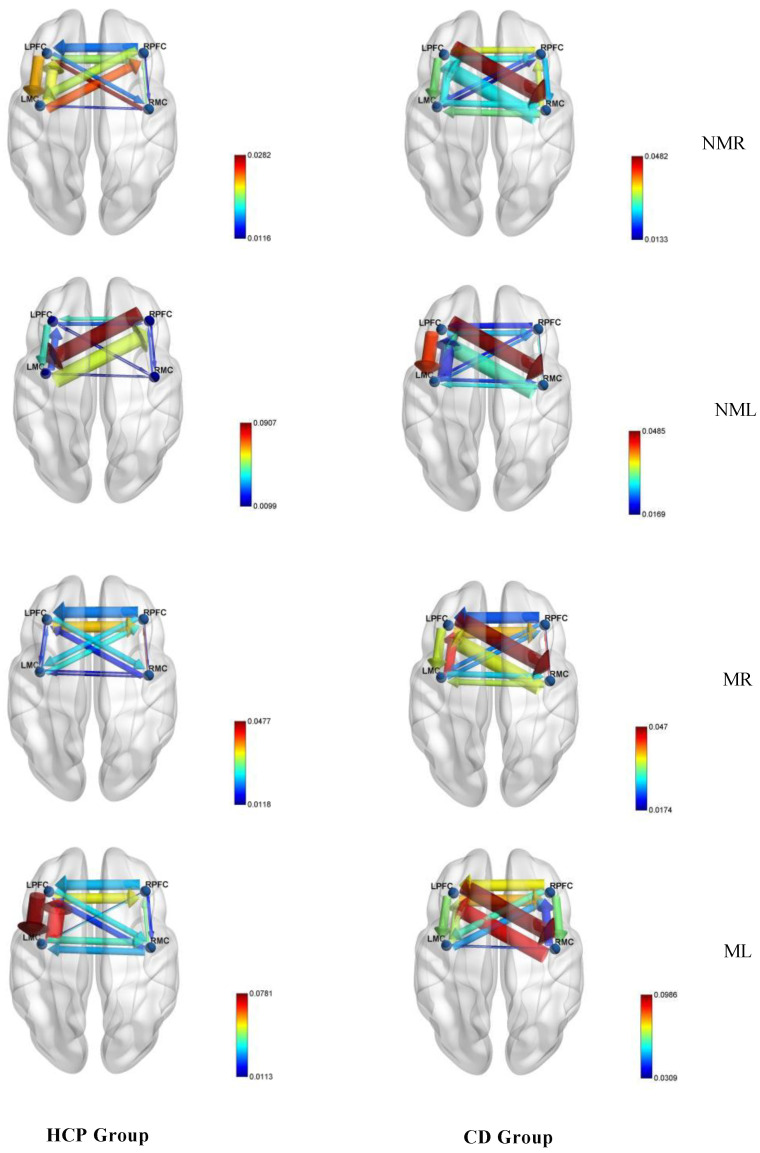
Effective connection mapping of GC values between the HCP group and the CD group ROI for different tasks. Among these, NMR de notes the normal vision right-hand task; NML denotes the normal vision left-hand task; MR denotes the mirror vision right-hand task; ML denotes the mirror vision left-hand task.

This study used conditional Granger causality analysis to systematically analyze the effective connectivity patterns between the HCP group and the CD group in four types of upper limb tasks. As shown in Figure 7, the overall average GC value matrix for both groups and the ROI connectivity map in Figure 8 (where arrow directions indicate information flow and line thickness reflects causal strength), both groups exhibit significant bidirectional connectivity but display task-dependent topological structural differences. In the HCP group, the NMR task formed three bidirectional pathways: LPFC ↔ RPFC, LPFC ↔ LMC, and RPFC ↔ LMC; while the NML group simplifies to bidirectional coupling between RPFC ↔ LMC and LPFC ↔ LMC. In the mirror vision task, the MR group exhibits interhemispheric interaction between LPFC ↔ RPFC and LPFC ↔ RMC, while the ML group maintains the core pathways between LPFC ↔ RPFC and LPFC ↔ LMC. In contrast, the CD group exhibited a more compact connectivity architecture in non-mirror tasks: in the NMR group, only the LPFC ↔ RMC bidirectional pathway was significant, while in the NML group, it expanded into a dual network of LPFC ↔ RMC and LPFC ↔ LMC; in mirror tasks, widespread coordination was activated: the MR task formed a ternary network of LPFC ↔ RMC, LPFC ↔ LMC, and LPFC ↔ RPFC; the ML task further develops into a triangular coupling system involving LPFC ↔ RPFC, LPFC ↔ RMC, and RPFC ↔ RMC.

#### 3.2.2. Coupling Strength

**Figure 9:**
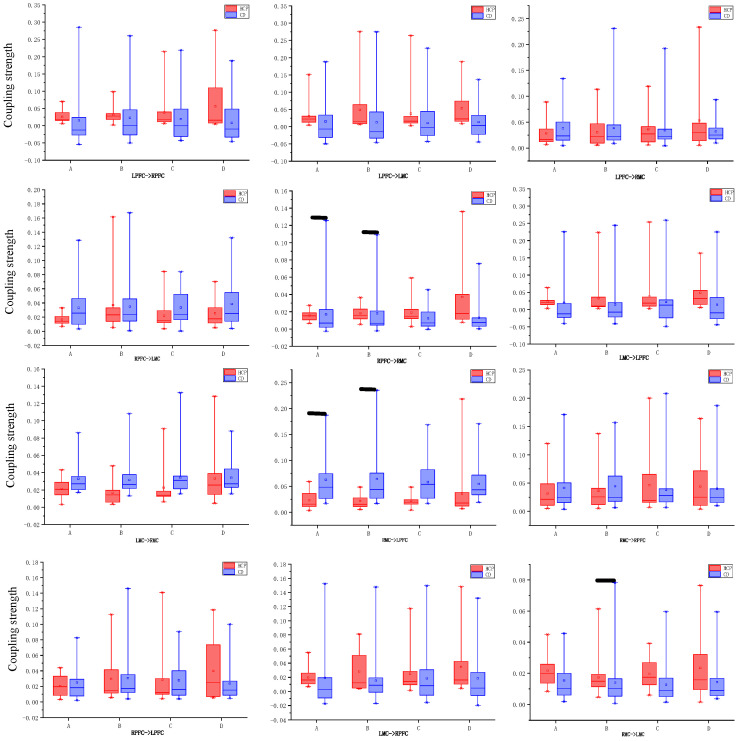
Box plots showing changes in task-related coupling strength in various directions between the HCP group and the CD group. The black line indicates *p* < 0.05.

Using an independent samples *t*-test, the system compared intergroup differences in effective connectivity between brain regions in the HCP group and the CD group under four visual-motor conditions. The analysis focused on directed connections between the prefrontal cortex (LPFC, RPFC) and the motor cortex (LMC, RMC). Statistical results indicated that abnormal connectivity patterns exhibit condition dependence and connection specificity. As shown in Figure 9: Under normal visual conditions, the RPFC → RMC connection exhibited significant intergroup differences in both NMR (right-hand tapping: t = −2.491, *p* = 0.018) and NML (left-hand tapping: t = −2.386, *p* = 0.023); Another significant abnormal connection was RMC → LMC, which was also significant under both NMR (t = −2.395, *p* = 0.022) and NML (t = −2.112, *p* = 0.041) conditions. Notably, the mirrored visual condition (MR/ML) significantly altered the abnormal connection pattern: in the MR task, RMC → LPFC (t = −2.334, *p* = 0.025) became the only significant connection, whereas in the ML task, all connections showed no statistical differences (*p* > 0.05).

## 4. Discussion

At the cortical activation level, CD children exhibited a typical contralateral control pattern during normal visual tasks, a finding validated in the study by De Campos et al. [26]. In contrast, children with hypoplasia of the corpus callosum demonstrated task-dependent abnormal activation. During the non-dominant hand NML task, HCP children consistently exhibited low activation in the LPFC, suggesting potential dysfunction in prefrontal regulation. This is speculated to be associated with impaired motor planning abilities caused by damage to the corticospinal tract. Concurrently, structural MRI studies have demonstrated that HCP children frequently present with abnormalities in frontal white matter microstructure or cortical thickness changes, providing a neuroanatomical basis for understanding their functional deficits [27]. During the contralateral hand NML task, HCP children exhibited compensatory activation enhancement in the LMC, reflecting neuroplasticity-mediated functional reorganization, though its efficacy remains task-specific [28]. The PFC regulates cognition and attention [29], while the MC governs sensorimotor control [30]. The widespread cognitive impairments, sensorimotor dysregulation, and attention deficits observed in HCP children [31] may be closely linked to functional abnormalities in the PFC and MC. Compared to CD children, HCP children exhibit significantly heightened LMC activation intensity during NML tasks. This may reflect adaptive compensatory mechanisms of the intact neural system, recruiting ipsilateral motor cortex resources to partially compensate for impaired contralateral pathways and maintain motor output [32,33,34]. This finding aligns with the enhanced compensatory activation in the lower limbs observed by Sukal-Moulton et al. in studies of brainstem cerebellar palsy (BCP) patients [24].

At the brain network level, Granger causality analysis based on fNIRS signals revealed bidirectional functional connectivity in both HCP children and CD children during motor states, consistent with the fundamental mechanism of multi-brain region collaboration in motor control [35]. During normal visual tasks, HCP children exhibited significantly weaker connectivity in both RPFC → RMC (right prefrontal cortex to right motor cortex) and RMC → LMC (right motor cortex to left motor cortex) pathways compared to CD children. These connections are closely associated with motor control functions, and their attenuation may represent the neural basis for upper limb ataxia in HCP children. Multiple studies using structural MRI and diffusion tensor imaging (DTI) further indicate structural alterations in brain regions involved in motor planning and execution (e.g., prefrontal cortex and motor cortex) in HCP children, providing a potential anatomical basis for the aforementioned abnormal functional connectivity [36,37]. However, mirrored visual tasks partially modulated these connectivity abnormalities: intergroup differences were observed only in RMC → LPFC feedback connections during MR tasks, suggesting visual reversal may influence motor output planning by enhancing feedback loops [38]. Under the ML task, all connectivity differences disappeared, suggesting that non-dominant hand movements in a mirrored environment may trigger more effective network compensation. This phenomenon corroborates the compensatory value of bilateral training for interhemispheric communication, providing direct evidence for the neurorehabilitation mechanism underlying mirror therapy.

Additionally, this study has some limitations. The small sample size and the fact that it primarily consisted of children with mild functional impairments (GMFCS Level I) limited the analysis of severity differences, statistical power, and generalizability. Furthermore, the spatial resolution of fNIRS was insufficient to effectively monitor deep brain regions related to motor control. Future studies should expand the sample to include children with varying degrees of severity, integrate multimodal imaging to enhance spatio-temporal resolution, introduce multidimensional behavioral indicators, and develop tasks with greater functional and ecological validity.

## 5. Conclusions

This study used fNIRS to analyze brain function during upper limb mirror training tasks in children with cerebral palsy and CD children. It validated the potential of mirror therapy to promote neural remodeling by regulating interhemispheric communication. This provides a theoretical basis for developing precision rehabilitation strategies targeting “brain network hub remodeling,” driving the transformation of cerebral palsy rehabilitation toward an evidence-based neuroplasticity intervention paradigm. Future rehabilitation strategies should shift from “symptom relief” to precision interventions based on “brain network hub remodeling.”

## Figures and Tables

**Table 1:** Characteristics of the subjects.

Groups	Number of Males	Age (M ± SD)	Height (M ± SD)	Weight (M ± SD)	GMFCS
HCP (*n* = 14)	6	8.34 (1.495)	140.55 (17.02)	37.341 (16.5214)	I
CD (*n* = 28)	14	7.5 (2.767)	115.5 (40.319)	44.186 (37.1998)	N/A

**Table 2:** fNIRS region of interest segmentation.

Area of Interest (ROIs)	fNIRS Channel
left frontal lobe, LPFC	7–10, 16
right frontal lobe, RPFC	15, 17, 21–24
left motor area, LMC	1–6, 11
right motor area, RMC	12–14, 18–20, 25–27

**Table 3:** Significant differences in beta values of ROIs in the four tasks of the HCP group after paired sample *t*-tests.

ROIs	NMR vs. NML			MR vs. ML			NMR vs. MR			NML vs. ML		
	M ± SD	t	*p*	M ± SD	t	*p*	M ± SD	t	*p*	M ± SD	t	*p*
LPFC	−1.008 ± 4.095	−0.921	0.374	−1.51 ± 3.252	−1.737	0.106	1.76 ± 5.574	1.181	0.259	1.258 ± 7.456	0.631	0.539
RPFC	−3.651 ± 11.942	−1.144	0.273	−2.421 ± 6.376	−1.421	0.179	0.418 ± 2.596	0.603	0.557	1.648 ± 9.462	0.652	0.526
LMC	−1.128 ± 2.764	−1.527	0.025 *	−1.033 ± 3.227	−1.197	0.253	1.177 ± 2.728	1.613	0.131	1.272 ± 4.713	1.01	0.331
RMC	2.685 ± 11.699	0.859	0.406	−1.674 ± 2.584	−2.425	0.031 *	0.912 ± 3.884	0.878	0.396	−3.447 ± 11.12	−1.16	0.267

Note: * indicates *p* < 0.05.

**Table 4:** Significant differences in beta values of ROIs in the four tasks of the CD group after paired sample *t*-tests.

ROIs	NMR vs. NML			MR vs. ML			NMR vs. MR			NML vs. ML		
	M ± SD	t	*p*	M ± SD	t	*p*	M ± SD	t	*p*	M ± SD	t	*p*
LPFC	0.72 ± 2.848	1.362	0.184	1.222 ± 5.187	1.269	0.215	−1.044 ± 4.999	−1.125	0.27	−0.543 ± 2.808	−1.041	0.307
RPFC	−1.405 ± 5.312	−1.424	0.165	−1.648 ± 4.085	−2.173	0.038 *	−1.851 ± 10.411	−0.957	0.347	−2.094 ± 11.523	−0.978	0.336
LMC	1.505 ± 9.193	2.317	0.028 *	0.618 ± 5.503	0.605	0.55	0.17 ± 4.422	0.207	0.837	−0.716 ± 10.36	−0.372	0.712
RMC	−0.244 ± 4.492	−2.068	0.048 *	0.336 ± 7.09	0.255	0.8	−1.237 ± 2.692	−2.474	0.02 *	−0.657 ± 7.563	−0.468	0.644

Note: * indicates *p* < 0.05.

## Data Availability

The original contributions presented in this study are included in the article. Further inquiries can be directed to the corresponding author.
